# Scrub Typhus Masquerading As Infectious Mononucleosis: A Case Report

**DOI:** 10.7759/cureus.85910

**Published:** 2025-06-13

**Authors:** Tomoko Yasuda, Takaaki Kobayashi, Kazuaki Aoki, Masayuki Nogi

**Affiliations:** 1 Department of General Internal Medicine, Kameda Medical Center, Kamogawa, JPN; 2 Department of Internal Medicine, University of Iowa Hospitals and Clinics, Iowa City, USA; 3 Department of Infectious Diseases, Kameda Medical Center, Kamogawa, JPN

**Keywords:** emerging infectious disease, mononucleosis-like, orientia tsutsugamushi, scrub typhus, tick-borne disease

## Abstract

Scrub typhus is a tick-borne disease caused by the intracellular organism *Orientia tsutsugamushi*. It typically presents with the cardinal "triad" of fever, rash, and eschar, along with other nonspecific symptoms. We report a case of scrub typhus in a 74-year-old man who did not exhibit the typical rash. He presented to the emergency room with a one-week history of generalized symptoms, including fever, throat pain, and myalgia, and was admitted due to suspected cholangitis based on elevated liver enzymes. However, computed tomography (CT) and magnetic resonance cholangiopancreatography (MRCP) ruled out this diagnosis. A transient rash developed after the initiation of antibiotics, considered to be a drug reaction. Further laboratory workup showed mildly positive results for cytomegalovirus (CMV)-IgM, and subsequent tests revealed an elevation of atypical lymphocytes, leading to a misdiagnosis of acute CMV infection. During a subsequent physical examination, an initially overlooked eschar was identified on his medial malleolus. Serology tests showed highly elevated *Orientia tsutsugamushi* IgM and IgG levels and treatment with tetracycline led to full recovery. Paired serology after two weeks showed no elevation in CMV antibodies, and the initial positive CMV-IgM result was considered insignificant. Scrub typhus can manifest with a wide range of symptoms, underscoring the importance of a thorough physical examination and maintaining clinical suspicion, especially in febrile patients in endemic areas.

## Introduction

Scrub typhus is a vector-borne disease endemic to the Asia-Pacific region, caused by the intracellular organism *Orientia tsutsugamushi *and transmitted by ticks and mites. An estimated one million people are infected worldwide each year, but the true incidence remains unknown due to substantial under-recognition and frequent misdiagnosis, including in Japan ​[1,2]​. While fever, rash, and eschar constitute the classical clinical "triad," symptoms are often wide-ranging and non-specific, mimicking other febrile diseases. Both diagnosis by serological testing and vaccine development have been complicated by the pathogen’s genetic diversity ​[3]​. Prompt recognition and treatment are critical, as delayed therapy can result in severe complications, including death ​[1]​. We report a case of a 74-year-old man with scrub typhus in whom diagnosis and treatment were delayed due to the lack of a characteristic rash and a clinical course mimicking cytomegalovirus (CMV) mononucleosis. This case highlights the diagnostic challenges of scrub typhus, especially when typical features are absent or serological results are confounded. 

## Case presentation

A 74-year-old male presented to the emergency department with a one-week history of fever and chills. Associated symptoms included bilateral lower leg myalgia, xerostomia, headache, diarrhea, nausea, pharyngeal pain, and loss of appetite. He denied other respiratory symptoms, rash, abdominal pain, muscle wasting, night sweats, or significant weight loss. He had not sought medical attention for the past 20 years, except for three dentist appointments in the past month for dental bridges. Otherwise, he was allegedly healthy, took no medications, and frequently took long walks in nearby forests. He reported having unprotected sexual intercourse with a commercial sex worker one week prior to the onset of fever. He denied alcohol consumption, recreational drug use, and international or domestic travel. 

At the time of presentation, his blood pressure was 134/75 mmHg, heart rate 110 beats per minute, respiratory rate 16 breaths per minute, peripheral oxygen saturation (SpO_2_) 96% on room air, and body temperature was 39.6°C. Physical examination was notable for bilateral posterior cervical lymphadenopathy but was otherwise unremarkable. There was no pharyngeal erythema, scleral icterus, palpable tenderness to abdominal or hepatic regions, decreased muscle tone, or motor weakness throughout. Skin findings lacked rashes, splinter hemorrhages, or petechiae. Laboratory workup revealed mild leukocytosis with neutrophilic predominance and no atypical lymphocytes (white blood cell count: 11,000/mcL; neutrophils: 86.0%; hemoglobin: 12.9 g/dL; platelets: 219,000/mcL). Liver enzymes were elevated, with aspartate transaminase (AST) at 150 U/L, alanine transaminase (ALT) at 153 U/L, alkaline phosphatase (ALP) at 495 U/L, gamma-glutamyl transferase (GGT) at 373 U/L, total bilirubin at 1.4 mg/dL, and direct bilirubin at 0.9 mg/dL. He was admitted with a provisional diagnosis of cholangitis, based on fever and elevated liver enzymes, and was started on intravenous ampicillin-sulbactam.

**Figure 1:**
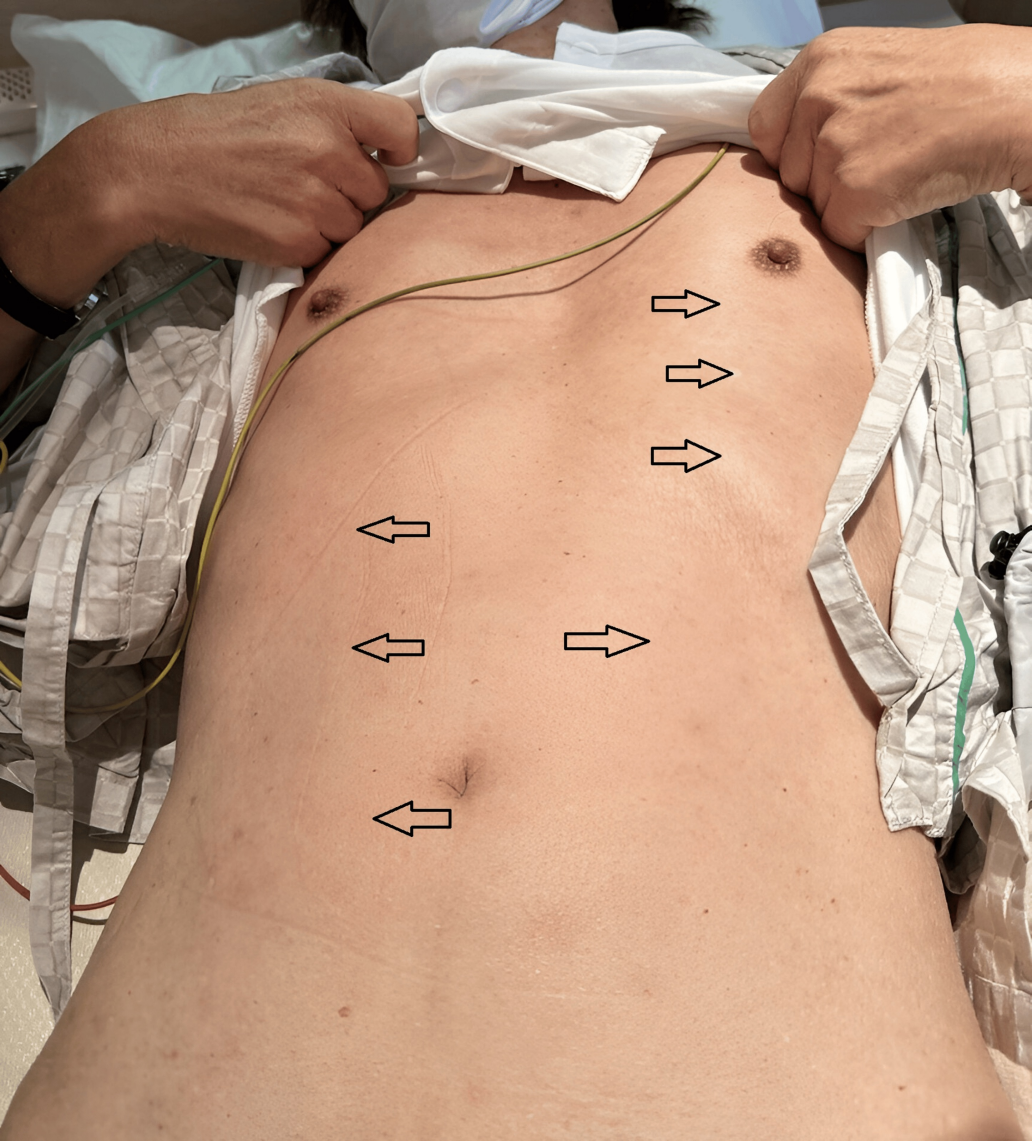
The transient rash A transient macular rash extending from the thighs to the trunk appeared after antibiotic use. The rash resolved shortly after discontinuation of the antibiotics and was considered a drug-induced rash. This picture was taken on the day the antibiotic was discontinued. Unfortunately, no photographs were taken during the active phase of the rash.

**Figure 2:**
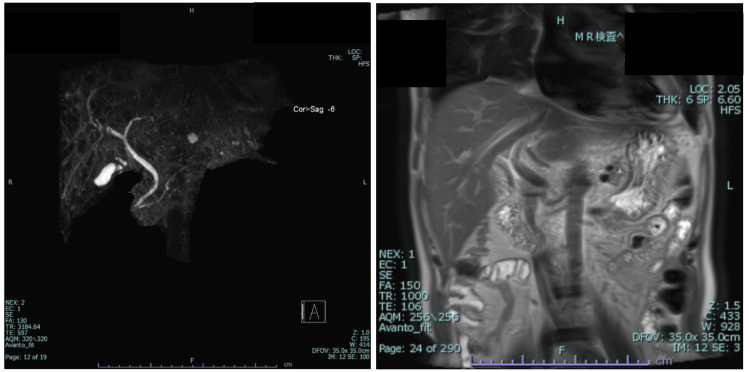
A coronal MRCP image MRCP revealed no abnormalities of the gallbladder or bile ducts, such as dilation, stenosis, stones, or masses, despite elevated liver enzymes. MRCP, magnetic resonance cholangiopancreatography

The following day, he developed a nonpruritic, erythematous rash from his abdomen to his thighs, which completely resolved immediately after antibiotic discontinuation (Figure 1). This rash was non-specific and considered to be an antibiotic-related drug rash. Computed tomography (CT) revealed several enlarged submandibular and cervical lymph nodes bilaterally without intraoral or cervical abscesses and a collapsed gallbladder with a single gallstone measuring 4×9 mm in size. There was no evidence of choledocholithiasis, or intrahepatic or extrahepatic bile duct or pancreatic duct dilation. Magnetic resonance cholangiopancreatography (MRCP) was likewise negative for biliary tract stenosis or dilation (Figure 2). The transthoracic echocardiograph was negative for valvular vegetations, mitral regurgitation, cardiac aneurysms, or ventricular wall motion asynergy. An abdominal ultrasound detected an 11 mm sized hepatic cyst on the S3 region but otherwise revealed no hepatic masses, surface irregularities, or hepatosplenomegaly. Blood cultures, respiratory multiplex polymerase chain reaction (PCR) assay (BioFire Film array system; BioFire Diagnostics), human immunodeficiency virus (HIV) antigen, hepatitis B and C (HBV and HCV) serology and antigens, treponema pallidum hemagglutination (TPHA), and rapid plasma reagin (RPR) were all unremarkable. Epstein Barr virus (EBV) showed past infection patterns, and CMV-IgM and IgG tested positive (Table 1). Atypical lymphocytes were detected on days 3 and 6 of admission at 4.0% and 3.0%, respectively, with lymphocytic predominance appearing on day 6. Due to diffuse lymphadenopathy, elevated liver enzymes, emergence of atypical lymphocytes, flu-like symptoms, and a positive CMV-IgM, he was suspected to have an acute CMV infection. Antiviral treatment was not started in accordance with the recommended mainstay management for CMV mononucleosis in an immunocompetent host without signs of end-organ damage. 

**Table 1:** Laboratory data: changes in laboratory results, including complete blood counts and liver function tests, over time WBC: white blood cells; neutro: neutrophil; lymph: lymphocyte; at-lymph: atypical lymphocyte; Hb: hemoglobin; Plt: platelet; AST: aspartate transaminase; ALT: alanine transaminase; LD: lactate dehydrogenase; ALP: alkaline phosphatase; GGT: gamma-glutamyl transferase; T-bil: total bilirubin; D-bill: direct bilirubin; CRP: C-reactive protein The day "number" refers to the day of admission as day 1.

Parameter	Day 1	Day 3	Day 6	Day 10	Reference
WBC (10^2^/mcL)	110	68	44	57	33-86
Neutro (%)	86.0	72.0	45.0	55.6	42.4-75.0
Lymph (%)	10	21	42	34.4	16-49
At-lymph (%)	0.0	4.0	3.0	0.0	0
Hb (g/dL)	12.9	12.0	12.2	12.6	11.6-14.8
Plt (10^4^/mcL)	21.9	20.9	33.7	46.4	15.8-34.8
AST (U/L)	150	153	102	40	13-30
ALT (U/L)	153	136	112	73	7-23
LD (U/L)	346	336	228	208	124-22
ALP (U/L)	495	417	347	286	38-113
GGT (U/L)	373	351	338	314	9-32
T-bil (mg/dL)	1.4	0.8	0.7	0.9	0.4-1.5
D-bil (mg/dL)	0.9	0.5	0.4	0.4	0.0-0.4
CRP (mg/dL)	20.75	10.93	2.52	0.40	0.00-0.14

**Figure 3:**
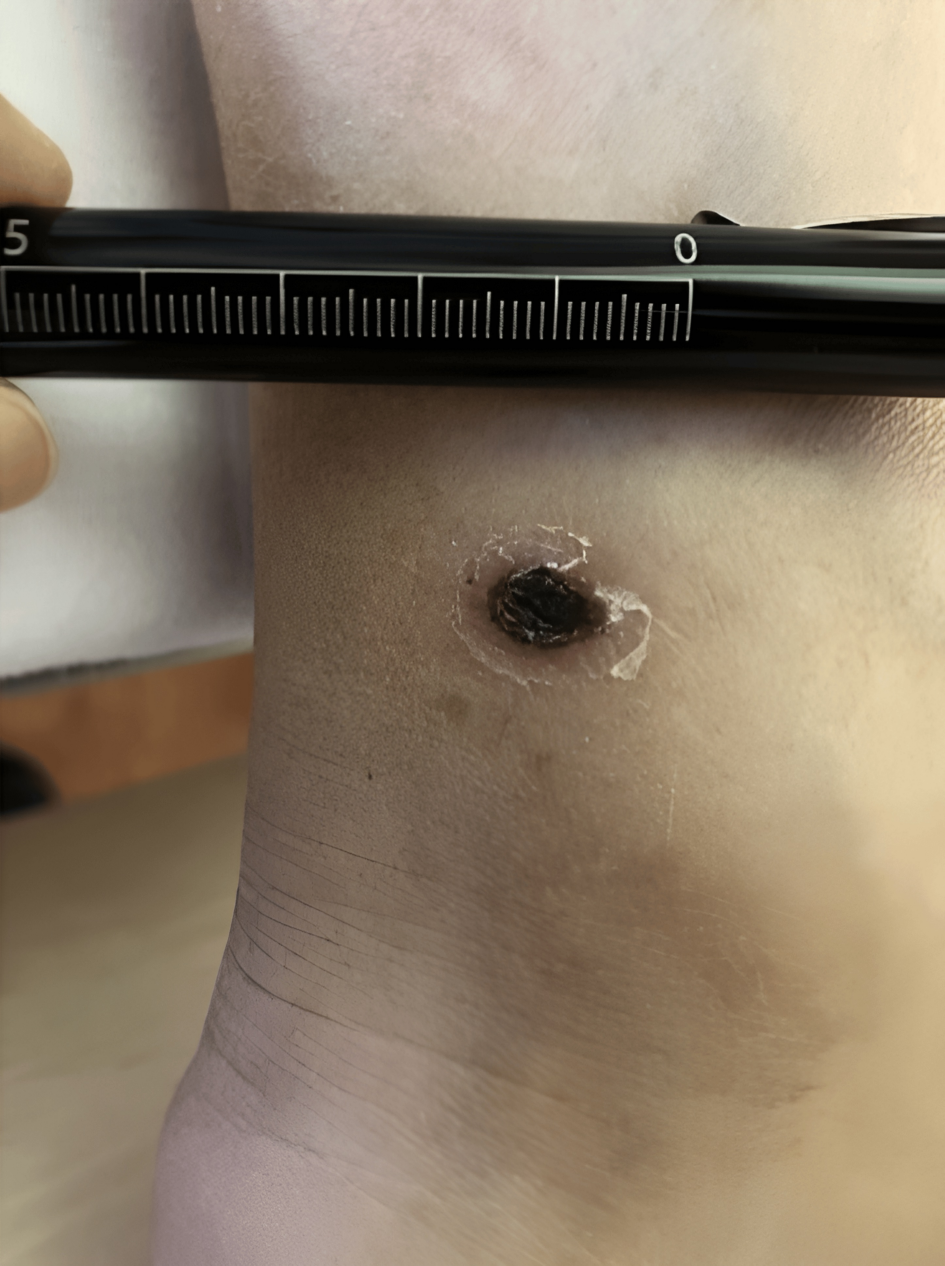
The eschar An eschar approximately 0.8x0.5 cm in size was discovered on the left medial malleolus.

However, the patient continued to have intermittent fever after admission. A thorough full-body examination was repeated on hospital day 4, revealing an eschar approximately 8×5 mm in size on the left medial malleolus, concealed under his socks (Figure 3). It had been overlooked in previous physical examinations, although it was discovered the patient himself had been aware of it prior to admission and had thought the scab was from “an ill-fitting shoe.” Rickettsial infection was suspected, and serum antibody tests and an eschar tissue biopsy were immediately submitted. However, initiation of antibiotic treatment was withheld due to a gradually improving clinical picture and uncertainty in diagnosis. 

On day 7 of admission, an indirect fluorescent antibody (IFA) serology test returned reporting significantly elevated *tsutsugamushi* IgM levels (Table 2), while the PCR test of the eschar returned negative for *Rickettsia japonica*. Oral doxycycline was initiated on day 7 for the persistent symptoms and administered for seven days. He was discharged on day 10 and upon his three-week follow-up visit, he showed complete resolution of symptoms and laboratory abnormalities including liver enzyme tests, with no further complications. The repeat CMV paired serology testing was negative, and a follow-up test for *tsutsugamushi* was deemed unnecessary due to the initial high IgM titer confirming his diagnosis. 

**Table 2:** Serologic tests for antibodies: Paired antibody testing was performed for CMV and HIV. Tsutsugamushi serology was performed once, as paired testing was deemed unnecessary for diagnosis based on initial IgM levels and the clinical syndrome CMV: cytomegalovirus; IgM: immunoglobulin M; IgG: immunoglobulin G; HIV: human immunodeficiency virus; CLIA: chemiluminescence immunoassay

Test	Day 1	Day 4	Day 20
CMV-IgM	Positive	-	Inconclusive
Index (reference: 0.01-0.99)	1.01	-	0.86
CMV-IgG	Positive	-	Positive
Index (reference: 0.00-5.99)	153	-	183
HIV 1/2 CLIA	Negative	-	Negative
Index (reference: 0.00-0.99)	0.100	-	0.100
*Tsutsugamushi* antibody (reference: 0-10)			
IgM Kato		2560	
IgG Kato		640	
IgM Karp		10>	
IgG Karp		160	
IgM Gilliam		2560	
IgG Gilliam		640	

## Discussion

Scrub typhus is a mite-borne disease endemic to the Asia-Pacific region, caused by *Orientia tsutsugamushi* of the Rickettsiaceae family. An estimated 1 million people are infected worldwide annually, with the death toll reaching as high as 150,000 per year ​[1]​. Over 400 cases are reported every year in Japan alone, although numbers are suspected to be vastly under-reported with many cases passing undiagnosed ​[2]​. Multiple subtypes with varying genome sequences have been identified, hindering the development of an effective vaccine ​[3]​. Its increasing prevalence, at times attributed to global climate change expanding the field of vector activity ​[4,5]​, has prompted the World Health Organization (WHO) to identify it as one of the most neglected causes of tropical fevers in terms of research, diagnosis, and control ​[6]​. 

Symptoms of scrub typhus appear after an incubation period of 10-12 days and are often flu-like, making it difficult to diagnose. In Japan, fever and rash are the most frequently observed features, present in 95% and 86% of all cases, respectively, although these figures widely vary worldwide ​[2,7,8]​. Other non-specific symptoms include malaise, headache, myalgia, nausea, abdominal pain, and cough ​[2,9]​. If left untreated, scrub typhus can proceed to cause systemic vasculitis with complications including meningoencephalitis, myocarditis, interstitial pneumonia, acute respiratory distress syndrome, renal failure, and disseminated intravascular coagulopathy ​[1,2,10]​. 

*Tsutsugamushi *infection can be tested directly or indirectly, although the latter method of detecting antibodies via agglutination reactions or immunofluorescence remains the mainstay in Japan, given that PCR testing for *tsutsugamushi *is not covered by national insurance. Serology tests can diagnose scrub typhus if there is a greater than four-fold increase in IgG antibody titers at acute and convalescent stages (at least 14 days apart), or in a single test based on elevated IgM in the appropriate clinical setting ​[11,12]​. Routinely tested antibodies include subtypes Karp, Kato, and Gilliam, although various strains show different patterns of reactivity ​[3]​. In this case, the eschar, highly elevated initial single timepoint IgM, and clinical syndrome that improved with doxycycline administration were interpreted to be sufficient for diagnosis for scrub typhus (although not to the specific subtype), thus a follow-up serology was not performed. Furthermore, symptoms had completely resolved in the two-week minimum interval required between paired tests, making the costly antigen test clinically inconsequential. Direct methods of testing include culture and PCR, which are often only performed in specialized facilities. A PCR of the eschar is more sensitive than blood and can be used for diagnosis even with prior antibiotic use ​[11,13]​. The pathognomonic scab is reported in 85% of all diagnosed cases in Japan and appears at the site of a chigger bite, most frequently on the anterior chest wall, such as the axilla and inguinal regions ​[14]​. 

Many infectious diseases can be mistaken for scrub typhus due to their similar clinical presentations as febrile illnesses. Especially noteworthy are malaria, dengue, leptospirosis, *Salmonella typhi*, and other rickettsial diseases due to their overlap in geographical spread in endemicity ​[15]​. Of these infections, *Rickettsia japonica, *which causes Japanese spotted fever, is also endemic in Japan and presents almost identically to scrub typhus with fever, rash, and a localizing eschar at the site of the tick bite, posing a significant diagnostic challenge in the country. Suggested features of *tsutsugamushi *that help distinguish it from *Rickettsia japonica* include the eschar being slightly larger, the rash being more likely to spare the hands and/or plantar areas, and more prevalent in cooler seasons ​[16]​. Regardless, the two diseases are difficult to differentiate singularly based on clinical presentation, and laboratory tests are usually necessary. Fortunately, the treatment of all rickettsial diseases requires the same antibiotics, making appropriate management possible even if there is a delay in definitive diagnosis. 

Once diagnosed, treatment for scrub typhus is relatively simple, with either oral or intravenous antibiotic monotherapy of tetracycline, azithromycin, or chloramphenicol. There have also been studies suggesting combination therapy of intravenous doxycycline and azithromycin to improve outcomes in severe cases ​[1]​. Of note, in recent years, the use of chloramphenicol has declined due to its toxicity profile, including severe side effects such as aplastic anemia. With appropriate treatment, the mortality rate is estimated to be around 1.4%; if untreated, this value increases to 6-70% ​[1,10,17]​, emphasizing the gravity of prompt diagnosis and management to reduce fatality ​[1]​. 

The diagnosis of this case was initially implicated by the failure to detect the eschar and the lack of a characteristic rash. The rise in prevalence of the disease itself and international travelers has increased the risk of its expansion to previously non-endemic regions. The variability of manifestations, as discussed above, poses a particular diagnostic challenge, especially to clinicians unaccustomed to scrub typhus. However, clinical suspicion is critical for initiating appropriate antibiotics in a timely manner, underscoring the importance of raising awareness of the disease and being able to hold a degree of suspicion based on patient history. The eschar can act as a suggestive marker but is small and easily overlooked, such as in this case, accentuating the importance of a detailed full-body physical exam. 

The positive CMV-IgM with manifestations resembling infectious mononucleosis with atypical lymphocytes further complicated the diagnosis of this case. There has been a similar case reported where scrub typhus was misdiagnosed as CMV, although CMV-IgM was not elevated in the report ​[18]​. Moreover, there have been past studies detailing false-positive CMV-IgM results in IM-like syndromes; some studies show specificity as low as 72% ​[19]​. In the previous literature, antigen cross-reactivity or interfering substances, such as heterophile antibodies, were listed as potential causes for the false-positive results ​[19,20]​.

## Conclusions

In conclusion, this case highlights the diverse and non-specific presentation of scrub typhus, an emerging infectious disease with various clinical mimickers. It reiterates the value of performing a detailed physical exam, especially in endemic areas, in guiding accurate diagnosis. Additionally, it draws attention to the possibility of false-positive antibody results due to potential cross-reactivity between *Orientia tsutsugamushi *and other common viruses, emphasizing the need for careful interpretation of serological findings.
